# Annual Address

**Published:** 1897-04

**Authors:** George A. Maxfield

**Affiliations:** The President; Holyoke, Mass.


### ANNUAL ADDRESS.[1]

BY THE PRESIDENT, GEORGE A. MAXFIELD, D.D.S., HOLYOKE, MASS.

To the Members of the Massachusetts Dental Society:

Ladies and Gentlemen,—An examination of the programme prepared for this meeting yields a fair exposition of the scope and aims of this Society.

In this, the president's annual address, I desire to present various subjects for your consideration. The past year has been one of progress in dental science. Of the many valuable contributions to dental literature, that of Dr. J. V. Black deserves special mention. He has presented many new and valuable facts in regard to the characteristics of the teeth, also of filling-materials, and much practical good must result from these studies.

The use of cataphoresis for obtunding sensitive dentine, accord-

---

[1] Presented at the annual meeting of the Massachusetts Dental Society, June 3, 1896.

ing to the method introduced by Dr. H. W. Gillett, of Newport, is a decided advance. The labor involved and the long series of experiments, the results of which he has freely given to the profession, proves him a man of true professional instincts and places his name on the roll of honor.

### DENTAL EDUCATION.

One of the most important subjects demanding our attention at this time is that of dental education. The advance made in the art and science of dentistry requires to-day a broad and liberal education of those entering the profession. Some of the dental colleges have kept pace with this progress and have advanced the necessary qualifications required of the student before allowing him to matriculate. The majority of dental colleges are delinquent in this matter, and only advance their standard as they are driven to it. The most severe criticism of the law of this State, which requires an examination of every candidate for registration, comes from these colleges. The revelations that are occasionally made regarding dental colleges show the need of a reform. On February 24 last there appeared in the New York *Daily Tribune* this revelation regarding the New York College of Dentistry:

"THE DENTAL COLLEGE CHARTER.

"*A New One may be granted by the Board of Regents.*

"If the charter of the New York College of Dentistry is revoked by the Regents of the University of the State of New York, as it probably will be, a new charter will be granted and the college will be continued without any interruption. It is the object of those who applied for a new charter to place the management of the institution in the hands of a board of trustees, and to give the faculty a stated salary for the services which they perform. At present the receipts of the college are divided among the members of the faculty after the running expenses have been paid, and there seems to be no way under the present mode of management to reduce the debt, which is large. In their statement to the regents, the protesting trustees say that a resolution was adopted by the trustees who were managing the affairs, and that under this resolution the profits have been divided each year among the five professors. They say,—

"Under this resolution the profits of the college have been divided each year among the five professors, one-fifth to each. These professors, therefore, receive no fixed salaries, but take all the profits. It appears that no surplus can ever be accumulated by the corporation on the present basis, nor can any sinking fund be established to take care of the existing bonded indebtedness. At the end of the last fiscal year the treasurer's report showed receipts of $62,297.52. This amount was disbursed as follows: $45,062.70 for salaries and wages; $16,876.81 for other expenditures, of which $5050 was for interest on debt, leaving in the treasury a balance of only $358.01."

The laws of the State of New York have taken this particular institution in hand and inaugurated a reform. On March 19 last the Board of Regents amended the charter of this college, and hereafter no person who is a salaried instructor, officer, or employé of the trustees in said institution shall after the issue of this amended charter hold the office of trustee or perform any act as such.

The grievous wrong done the dental profession by such institutions as this New York College of Dentistry has been cannot be comprehended. If rumors are correct, there are many other colleges that are conducted on the same basis and for the same object. Can we expect that the graduates of these institutions will be professional men while their teachers, by greed and avariciousness, debase the nobler aspirations that mark the difference between a trade and a profession? Some of the graduates of this and like institutions are an honor to the dental profession, but the credit belongs not to their Alma Mater, but is the result of the inherent qualities in the individual himself.

The only means by which the dental profession can be placed upon the plane it should occupy are in the hands of the dental colleges. As long as their principal object is to make money, we cannot find that degree of proficiency in their graduates that the times demand. It is time these institutions were placed on a different basis. There should be no more close corporations conducting dental colleges. Every dental college should be compelled to publish annually a financial statement, showing the amount of their receipts and how their money is disbursed, that the profession may know what part of the college income is expended for educational purposes.

The education of the dentist should be on a broad and liberal basis, and dental colleges should be conducted as are other educational institutions. It is said that three thousand dollars would be over rather than under the average salary paid the professors of our great universities, men who devote their whole time to their professorships. Many of these men would command a much higher income from some other profession or from a business, but their love for their work stands first, their income a secondary consideration.

Only such men as are imbued with a love of science should be appointed to the professorships in dental colleges. Then, and not until then, will the colleges fulfil their whole duty to their students.

The National Association of Dental Faculties is an endeavor in the right direction, but from the very nature of things they cannot cope with this gigantic evil that underlies the system. Only through the concerted action of dental societies will this reform be accomplished.

In regard to entrance examinations there is much need of reform. I quote from the announcement of a college that has been considered as one of the leading dental schools, "The applicant must be conversant with the English language and give satisfactory evidence of having received a good preliminary education." A personal knowledge of some students that have entered this college evidences the fact that the preliminary education required for entrance to the average high school is much too high a standard for those entering this college.

The State of New York has taken up this subject, and has enacted a law which went into effect August 1 last requiring every student before matriculating in a dental college to file a certificate of the regents that he has had a satisfactory preliminary education, which for those matriculating after January 1, 1897, shall be not less than a full high-school course. At graduation, before receiving a diploma and the degree of D.D.S., the student must pass an examination by the Dental Examining Board of the State. The conferring of diplomas is essentially placed in the hands of a board entirely independent of the teaching faculty. Hereafter this ought to assure a thorough course of instruction and a high standard in all students graduating from any dental college in that State.

In the last announcement of the Boston Dental College they reported one hundred and sixty-nine students in attendance, and I am informed by one of their trustees that the total amount expended last year for salaries—professors, demonstrators, janitors, and a nurse at a hospital—was $12,360. The New York College reported three hundred and sixty-three matriculates, fifty-seven of which did not attend lectures, leaving the number of students as three hundred and six. According to their trustees they paid $45,062.70 as salaries. With this comparison of these two institutions comment is unnecessary. I would recommend that this Society formulate an address, embodying the facts here presented, and appoint a committee to present it at the next meeting of the American Dental Association, and demand that they take some action in an endeavor to establish these needed reforms. The strong opposition that will arise at any attempt to reform these institutions should not deter us from attending to our duty.

### DENTAL LEGISLATION.

Dental legislation, or the enactment of laws regulating the practice of dentistry, has been an important factor in the progress and development of dental science. Nine years ago such a law was enacted in this State, and every candid mind will admit that during these years much good has been accomplished. This law possesses many valuable features, and at the time of its enactment was superior to that of other States. A marked effect of this law was the raising the standard of qualifications necessary for graduation by many of the dental colleges. As time passes the weakness of things become apparent, and we now recognize the defects of this law, and that the time has arrived when they should be remedied. Twice the Board of Registration has attempted to have some minor changes made in this law, but they were unsuccessful. That their attempts failed is not to be wondered at. How could they expect the Legislature would pass any amendments to this law when advocated by only five members of the profession,—the profession throughout the State not evincing the slightest interest in the matter?

I desire at this time to point out some of the defects of this law, and advise ways and means by which the necessary changes can be accomplished. First, there is no provision regarding the moral character of the person applying for registration. The law reads, "They shall be examined with reference to their knowledge and skill in dentistry and dental surgery," and if the examination prove satisfactory the board shall issue a certificate. Several years ago an inmate of the State prison (who since, in a neighboring State, was condemned for murder and died in prison) was granted a certificate by the board. For this they were severely criticised; this, also, was made the basis of an attack against the president of the board in an effort to have him removed. According to a strict interpretation of the law, the board could not have done otherwise. About two years ago a dentist, driven from a city in one of the Western States on account of fraudulent practices, came before the board under an assumed name. Acting under the advice of the attorney-general, the board issued him a certificate bearing the assumed name he had given them. At last accounts he was practising in this city of Boston in his own name. Other cases might be cited, but these are sufficient to show a radical weakness in the law to accomplish its main purpose. A clause specifying that a person applying for examination must be of good moral character would greatly relieve the board, and many unfit persons could not be registered.

There should be an age limit; candidates should be not less than twenty-one years of age.

The fee for examination should be increased, and the number of times a person can be re-examined should be limited to one or two; after that an additional fee should be charged for each examination. As it is now, the fee is only ten dollars, and no limit to the number of times a person can be re-examined. This does not furnish sufficient funds to pay the legitimate expenses of the board.

The option as to whether the examination shall be oral or written should be left for the board to decide. Under the existing law the candidate generally chooses the oral examination, thus obliging the members of the board to sacrifice too much time and labor to properly attend to the examinations.

A clause should be inserted requiring a candidate for examination to present sufficient evidence to the board that, prior to the commencement of his professional studies, he had a preliminary education equivalent to that required of students entering the dental colleges of this State.

Section 9 of the law should be stricken out. It reads, "Nothing in this act shall apply to any practising physician who is a graduate from the medical department of any incorporated college." I would offer the following as a substitute: "This act shall not be construed to prohibit an unlicensed person from performing merely mechanical work upon inert matter in a dental office or laboratory, or the student of a registered dentist from assisting his preceptor in dental operations while in the presence of and under the personal supervision of the instructor, or a duly registered physician from treating diseases of the mouth or performing operations in oral surgery. But nothing in the provisions of this act shall be construed to permit the performance of dental operations by any unregistered person under cover of the name of a registered practitioner." Under the present law it is a misdemeanor to allow a student to do anything whatever in an office except upon inert matter. In fact, a strict interpretation of the law, I fear, would not allow even this, if it was anything pertaining to the practice of dentistry.

Provision should be made for the appointment of a prosecuting officer, similar to that of the pharmacy law. Under present conditions there are constant violations of the law in nearly every city and town in the State. Many dentists are employing unregistered assistants, and others allow students to perform operations without any supervision. There is a general misunderstanding in regard to the dental law. Many believe, as did ex-Governor Long, that it is for the protection of the dentist from competition, confining the practice of dentistry to a privileged few. This important fact should be emphasized and re-emphasized, that the dental law is for the benefit of the whole people, to protect the public from incompetent practitioners.

It is the duty of this Society to see that the dental law is sustained, and to use its influence to bring about the needed amendments. I would recommend that this Society annually appoint a committee on dental legislation. The old saying that "What is every one's business is no one's business" appears to be the present condition. In attempting this work there must be some one to take the lead, and there must be a reserve force that can be called into action in time of need. If this Society will undertake this work in a proper and systematic manner, there can be no doubt of the result.

If this committee should present a bill to the Legislature to amend the present law, it would be their duty to keep track of its progress, to urge an attendance of the members at the legislative committee's hearing, and, when the bill is reported to the Legislature, then every member should be notified and directed how to use his influence with the senator and representatives from his district for the passage of the bill. If at any time adverse dental legislation should be attempted, this committee will bring the whole influence of this Society against such measures.

Two years ago the medical profession secured the passage of a law to regulate the practice of medicine. This law needs amending, to make it of any force or benefit to the people. Thus far the Board of Registration in Medicine has shown a narrow spirit in dealing with those members of the dental profession who desired to register under their law, and usurped powers that properly belong to the courts of law. An attempt will soon be made to amend this law, and in this connection arises an important question, "Where can the line be drawn that separates the practice of dentistry from the practice of medicine?" While we should aid the medical profession in their endeavors to secure the passage of a good law, yet the spirit already manifested indicates the necessity of taking some measures to protect the interests of the dental practitioner. This will be properly attended to if this Society appoint a committee as suggested.

Last April, by the resignation of a member, a vacancy occurred on the Board of Registration in Dentistry. As a result of the efforts of this Society, through its committee, his Excellency Governor Wolcott appointed Dr. D. M. Clapp, of Boston, to fill the vacancy.

BOARD OF REGISTRATION IN DENTISTRY.

To properly attend to the duties required of each individual member of the Board of Registration in Dentistry demands a sacrifice of much time and labor. This Society should show its appreciation of this work, and I would suggest that at this meeting some action be taken, by resolution or otherwise, as an expression of its desire to co-operate and maintain the board in their efforts to elevate the standard of dentistry in this State.

And now as to the personal affairs of this Society. The reports from the different district societies, as presented to the councillors this morning, I briefly recapitulate here.

*Western District.*—Twenty-one members; meetings well attended, and everything in flourishing condition. The majority of dentists of Berkshire County are enrolled as members.

*Valley District.*—Twenty-eight members; fair interest manifested. Specially prepared programmes call out a good attendance.

*Central District.*—Fourteen members; fair interest maintained; prospects encouraging. There has been no attendance from outside the city of Worcester at any of the meetings.

*Southeastern District.*—Twelve members. No meetings have been held, and no report has been presented.

*Northeastern District.*—Nine members; have held no meetings. With the exception of two or three members, there is apparently no interest in this district.

*North Metropolitan District.*—Forty-one members; fair interest.

*South Metropolitan District.*—Seventy-eight members; fair interest.

Total membership, two hundred and three.

While these reports may not fulfil our expectations, yet I believe we have much to encourage us; our organization is more complete, and we are better able to take advantage of the opportunities presenting. The work of building up the district societies must necessarily be slow. If in formulating plans we truly recognize the conditions confronting us, we shall surely succeed.

There is a lamentable lack of professional tone and vigor, and one need hardly look below the surface to be able to trace the cause for this condition back to those money-making institutions that have posed as dental colleges. There is missionary work to be done, and the need most apparent is for a more personal responsibility by the individual members, and a willingness to sacrifice a little time and labor.

I would recommend that the four Eastern District Societies hold union meetings in Boston at least twice a year, taking an afternoon and evening; the programme, consisting of clinics, demonstrations, and papers, and a specially worded invitation, to be sent to all reputable dentists residing in these districts who are not members.

I would recommend the Central District Society to hold at least four meetings during the year, afternoon and evening, and to make special efforts to secure the attendance of those residing out of the city.

